# Supplementary material for: Long-Term Survival and Regeneration Following Transplantation of 3D-Printed Biodegradable PCL Tracheal Grafts in Large-Scale Porcine Models
Source: Bioengineering (Basel). 2024 Aug 14;11(8):832. doi: 10.3390/bioengineering11080832 (PMC11351403; doi:10.3390/bioengineering11080832)
Supplement: Supplementary file 1 [file bioengineering-11-00832-s001.zip › bioengineering-3137158 supplementary file.docx]

Article

Long-Term Survival and Regeneration Following
Transplantation of 3D-Printed Biodegradable PCL Tracheal Grafts in Large-Scale Porcine Models

Sen-Ei Shai ^1,2,3,^*^,†^, Yi-Ling Lai ^1,†^, Yi-Wen Hung ^4,5^, Chi-Wei Hsieh ^6^, Kuo-Chih Su ^7^, Chun-Hsiang Wang ^7^,
Te-Hsin Chao ^8^, Yung-Tsung Chiu ^9^, Chia-Ching Wu ^6,10^ and Shih-Chieh Hung ^11,12^

^1^ Department of Thoracic Surgery, Taichung Veterans General Hospital, Taichung 407219, Taiwan; windjay77@hotmail.com

^2^ Department of Applied Chemistry, National Chi Nan University, Nantou 545301, Taiwan

^3^ Institute of Clinical Medicine, National Yang-Ming Chiao-Tung University, Taipei 112304, Taiwan

^4^ Animal Radiation Therapy Research Center, Central Taiwan University of Science and Technology,
Taichung 406053, Taiwan; hongiw@yahoo.com.tw

^5^ Terry Fox Cancer Research Laboratory, Translational Medicine Research Center, China Medical University Hospital, Taichung 404327, Taiwan

^6^ School of Medicine, National Cheng Kung University, Tainan 701401, Taiwan;
peter100yahoo@gmail.com (C.-W.H.); joshccwu@mail.ncku.edu.tw (C.-C.W.)

^7^ Department of Medical Research, Three Dimensional Printing Research and Development Group,
Taichung Veterans General Hospital, Taichung 407219, Taiwan; kaoche2000@gmail.com (K.-C.S.); wangch@vghtc.gov.tw (C.-H.W.)

^8^ Division of Colon and Rectal Surgery, Department of Surgery, Chiayi and Wangiao Branch,
Taichung Veterans General Hospital, Chiayi 600573, Taiwan; thchao@vghtc.gov.tw

^9^ Department of Medical Research and Education, Taichung Veterans General Hospital,
Taichung 407219, Taiwan; ytchiu@vghtc.gov.tw

^10^ Department of Cell Biology and Anatomy, College of Medicine, National Cheng Kung University,
Tainan 701401, Taiwan

^11^ Integrative Stem Cell Center, China Medical University Hospital, Taichung 404327, Taiwan; hung3340@gmail.com

^12^ Institute of New Drug Development, China Medical University, Taichung 404328, Taiwan

***** Correspondence: sse50@yahoo.com; Tel.: +886-975-351-109

^†^ These authors contributed equally to this work.

**Supplementary Materials**


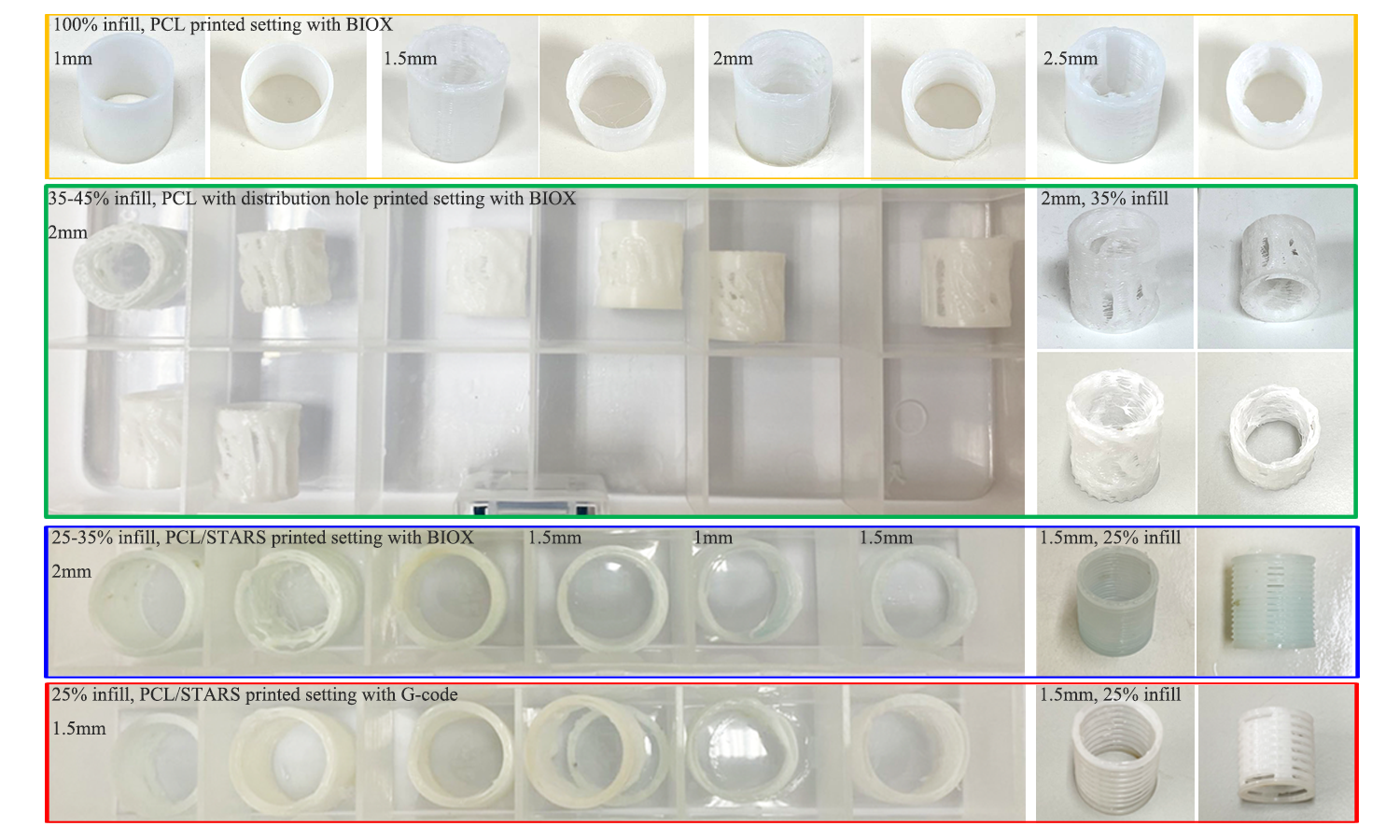


**Figure S1.** Various formulations of actual 3D-printed PCL-based tracheal grafts. (Panel 1, yellow) PCL tracheal grafts with 100% infill and a thickness of 1-2.5mm were configured and printed by BIOX. (Panel 2, green) PCL tracheal grafts with 25-35% infill and a thickness of 2mm, featuring irregular distribution holes, were configured and printed by BIOX. (Panel 3, blue) PCL tracheal grafts with 25-35% infill and a thickness of 2mm, equipped with regular distribution holes using STARS sacrificial material, were configured and printed by BIOX. (Panel 4, red) PCL tracheal grafts with 25% infill and a thickness of 1.5mm, incorporating regular distribution holes using STARS sacrificial material, were set using G-code and printed by BIOX.


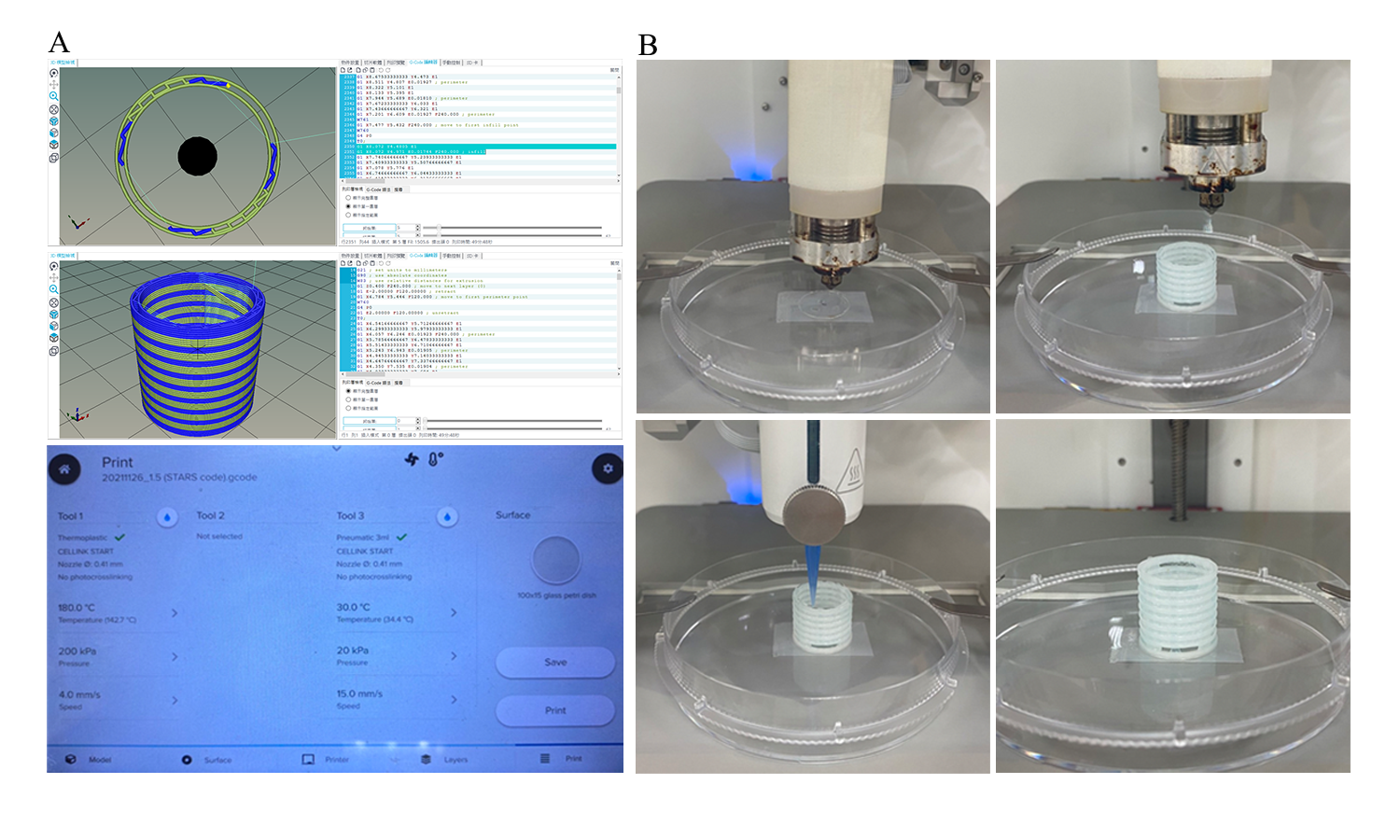


**Figure S2.** PCL tracheal grafts were printed by BIOX. Serial procedure of 3D printing PCL-based tracheal grafts with BIOX. (A, top) PCL tracheal grafts were designed using SolidWorks software and edited with G-code through Cellink Heart Ware software. (A, bottom) The printing parameters (pressure and speed) of the BIOX bioprinter were set. (B) Tool1 denotes the PCL cartridge; Tool3 refers to the "STARS" sacrificial material cartridge.


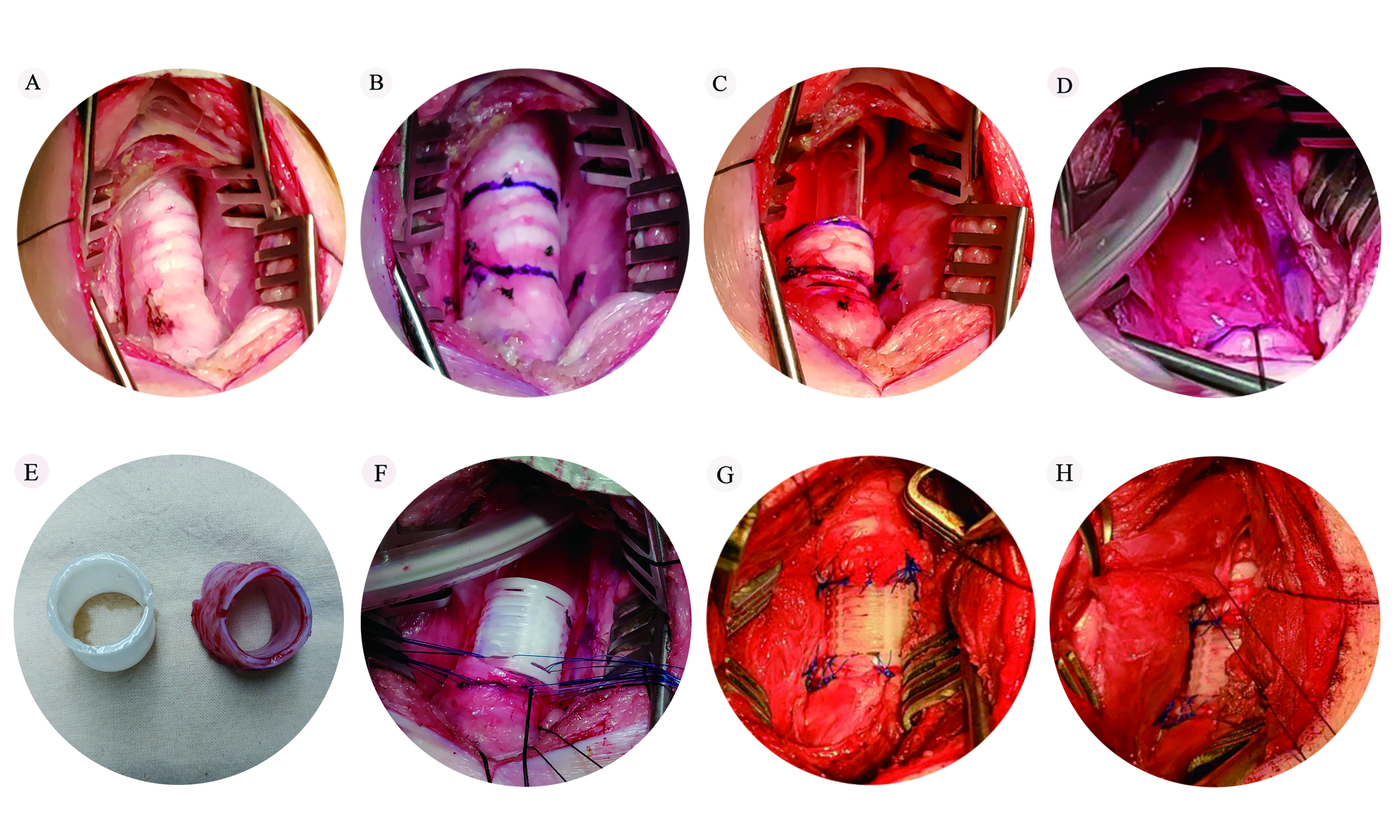


**Figure S3.** Procedures for surgical intervention for tracheal graft transplantation in a porcine model. (A-B) Marking a two cm long section of the native trachea for resection. (C-D) Removed tracheal segment. (E) Aseptic 3D-printed grafts in good condition were used as implants. (F) Anastomosis of the proximal end of the trachea with the graft. (G) Completion of graft reconstruction. (H) Distal trachea fixed to the strap muscle with a dual-end needle 3-O prolene for tension suture.


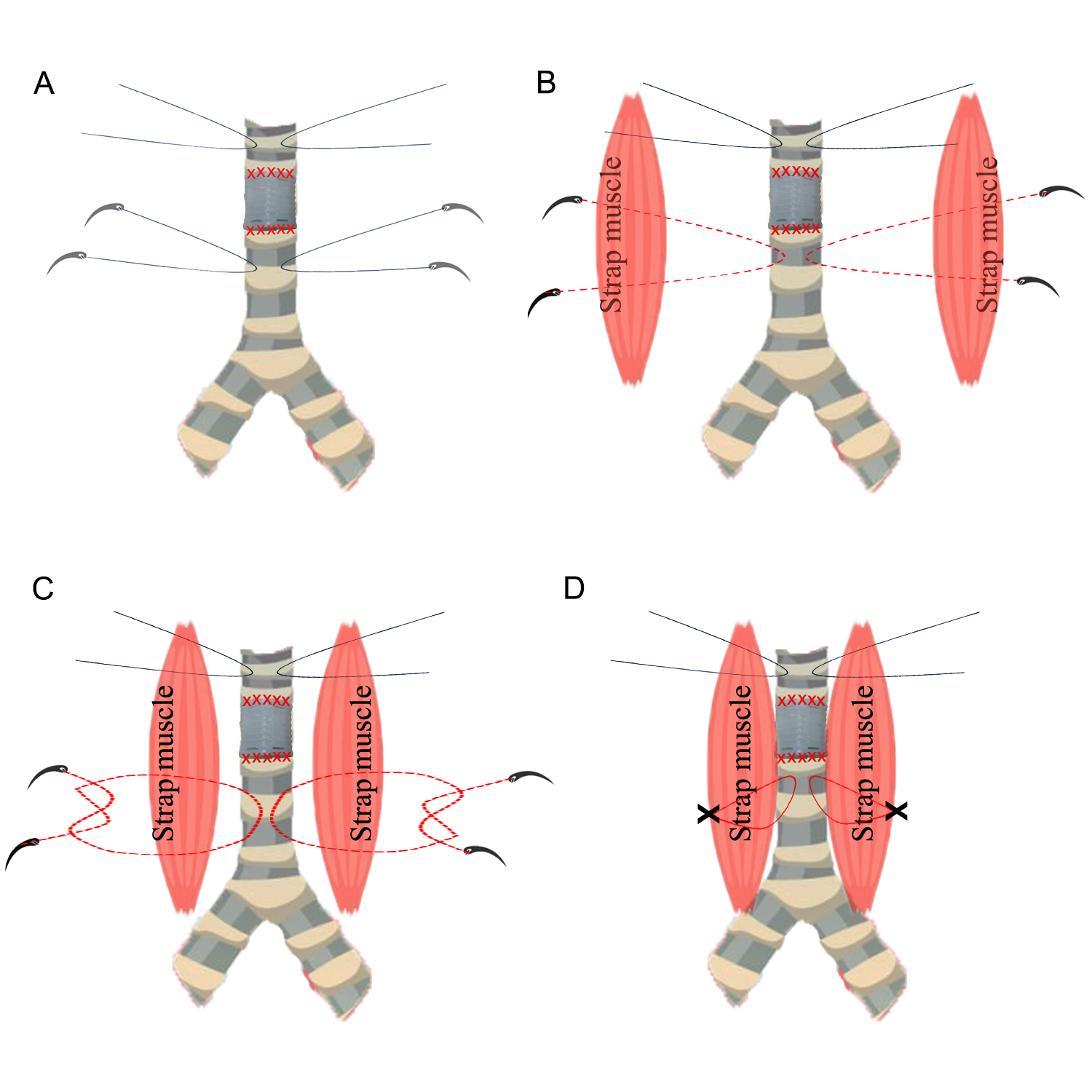


**Figure S4.** Method for fixation of the distal trachea to the strap muscle in Group II. (A) Traction sutures were applied to the bilateral proximal end with 3-O vicryl and to the distal end of the trachea with a dual-end needle 3-O prolene suture. (B) Sutures were passed through the strap muscle on both sides to release tension after graft implantation. (C) The sutures were tied to approximate the trachea and muscle. (D) The distal trachea was fixed to the strap muscle to counteract the forceful traction caused by thoracic negative pressure.


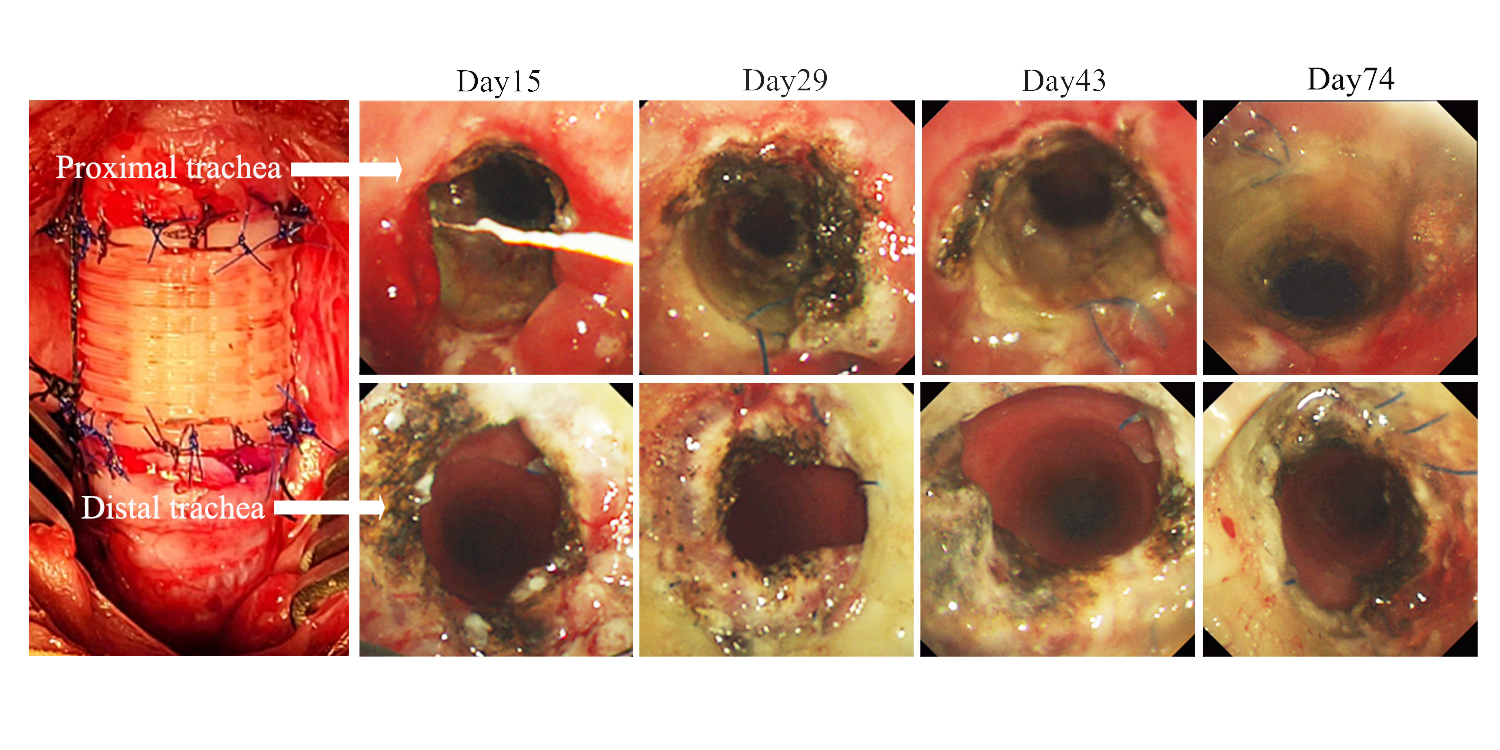


**Figure S5.** Bronchoscopy images of laser ablation for granulation tissue 15-74 days after PCL tracheal graft transplantation.


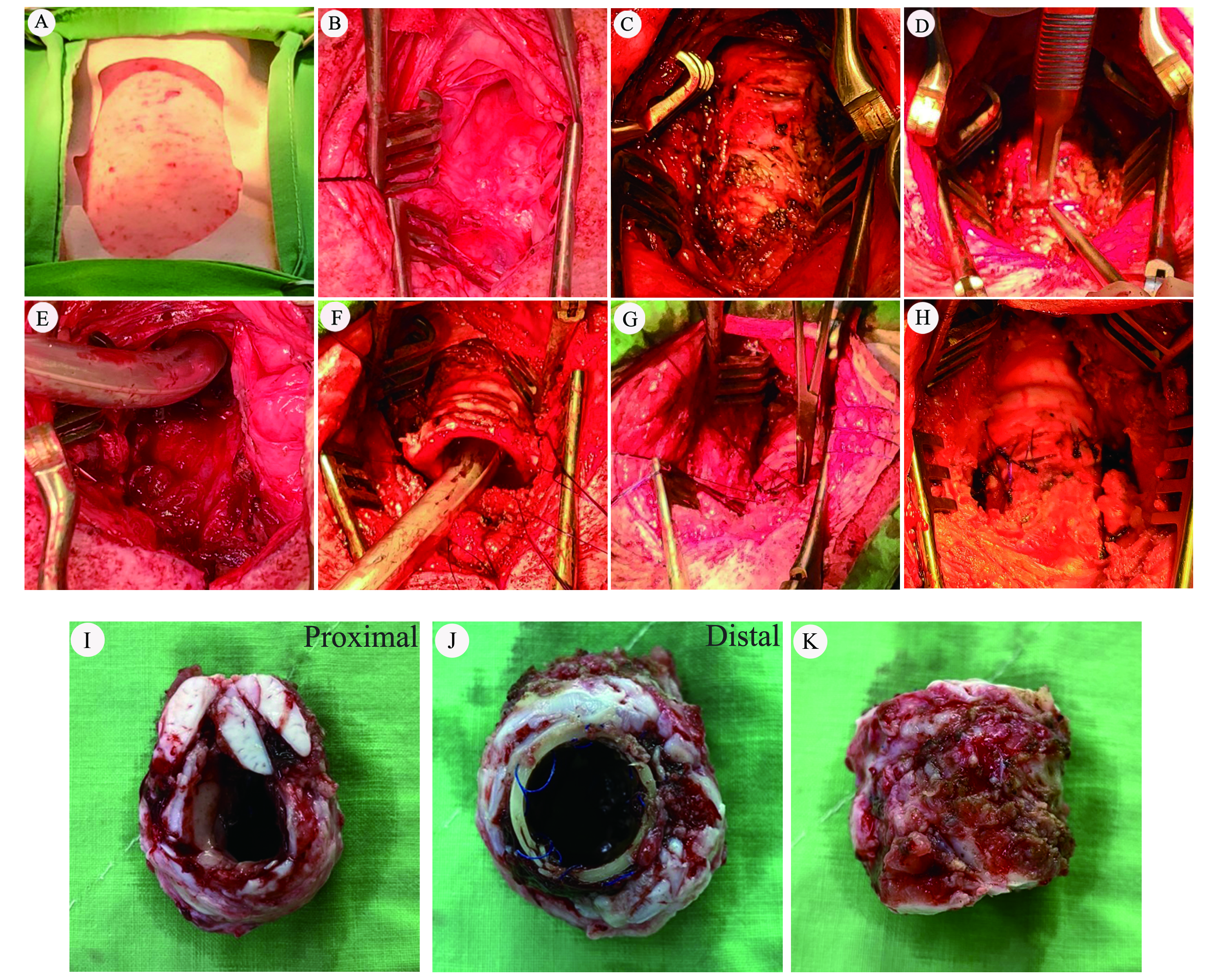


**Figure S6.** Harvested Cylindrical Soft Neotissue Growth Outside Tracheal Graft. (A-B) Well-healed wound without infection 92 days after graft transplantation. (C-E) Harvested cylindrical soft neotissue growth outside the tracheal graft with PCL graft. (F-H) Anastomosis of both ends of the trachea and completion of tracheal reconstruction. (I-K) Gross observation of cylindrical soft neotissue growth outside the tracheal graft with PCL graft.


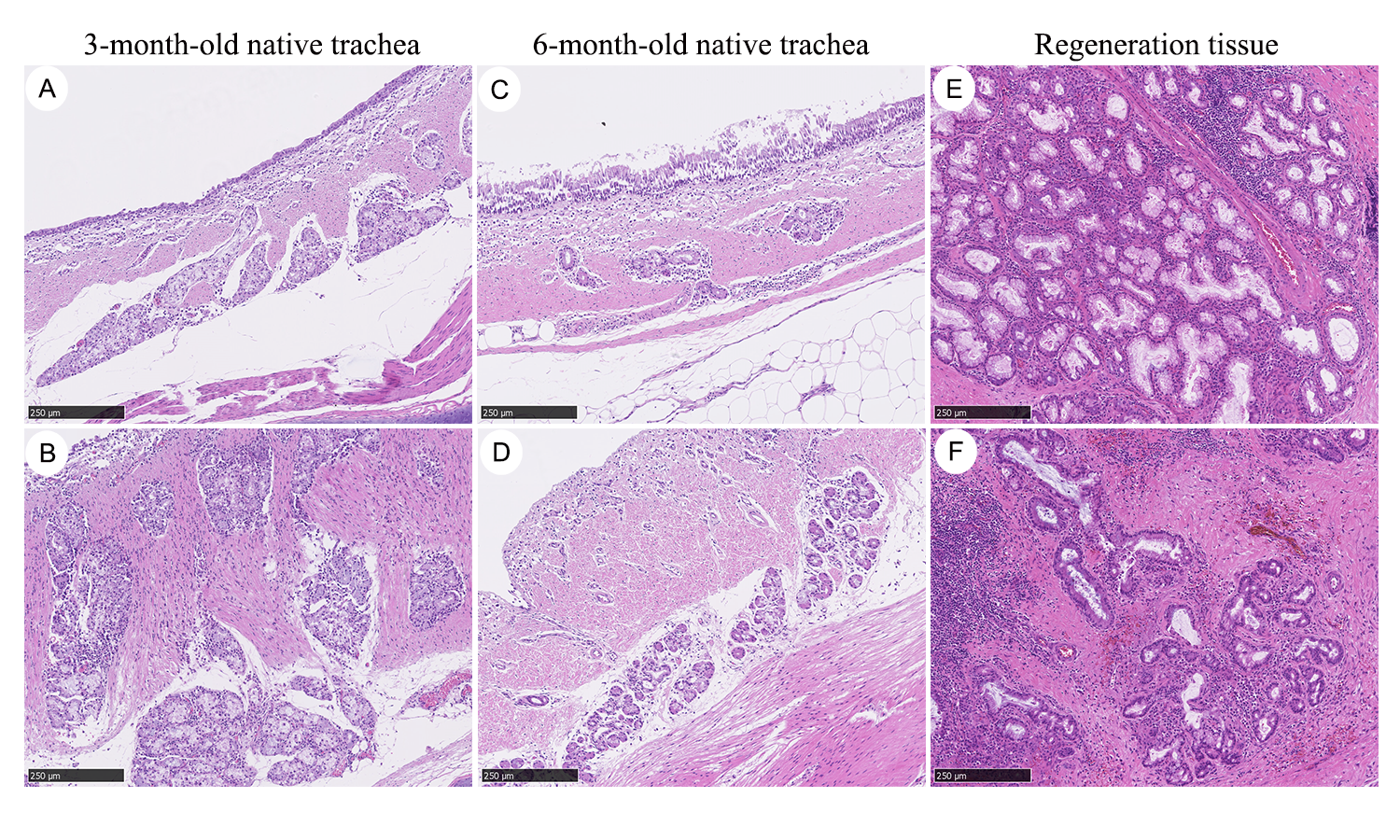


**Figure S7.** A Histological Comparison of Submucosal Glands from Native Porcine Tracheas at Different Ages and Regenerated Tracheal Tissue After a 90-Day Implantation Period. (A-B) Illustrate the submucosal glands from a 3-month-old porcine trachea, depicting a homogeneous distribution throughout the tissue. (C-D) Display the submucosal glands from a 6-month-old porcine trachea, which show a more heterogeneous distribution, suggesting developmental changes as the animal matures. (E-F) Highlight the submucosal glands in the regenerated tracheal tissue, which appear more abundant and larger in size compared to those in the native tracheal tissue. All images are magnified at 100x, providing a detailed view of the glandular structures and their distribution patterns. This comparison offers insights into glandular development in the native trachea with age and the differences observed in regenerative growth.


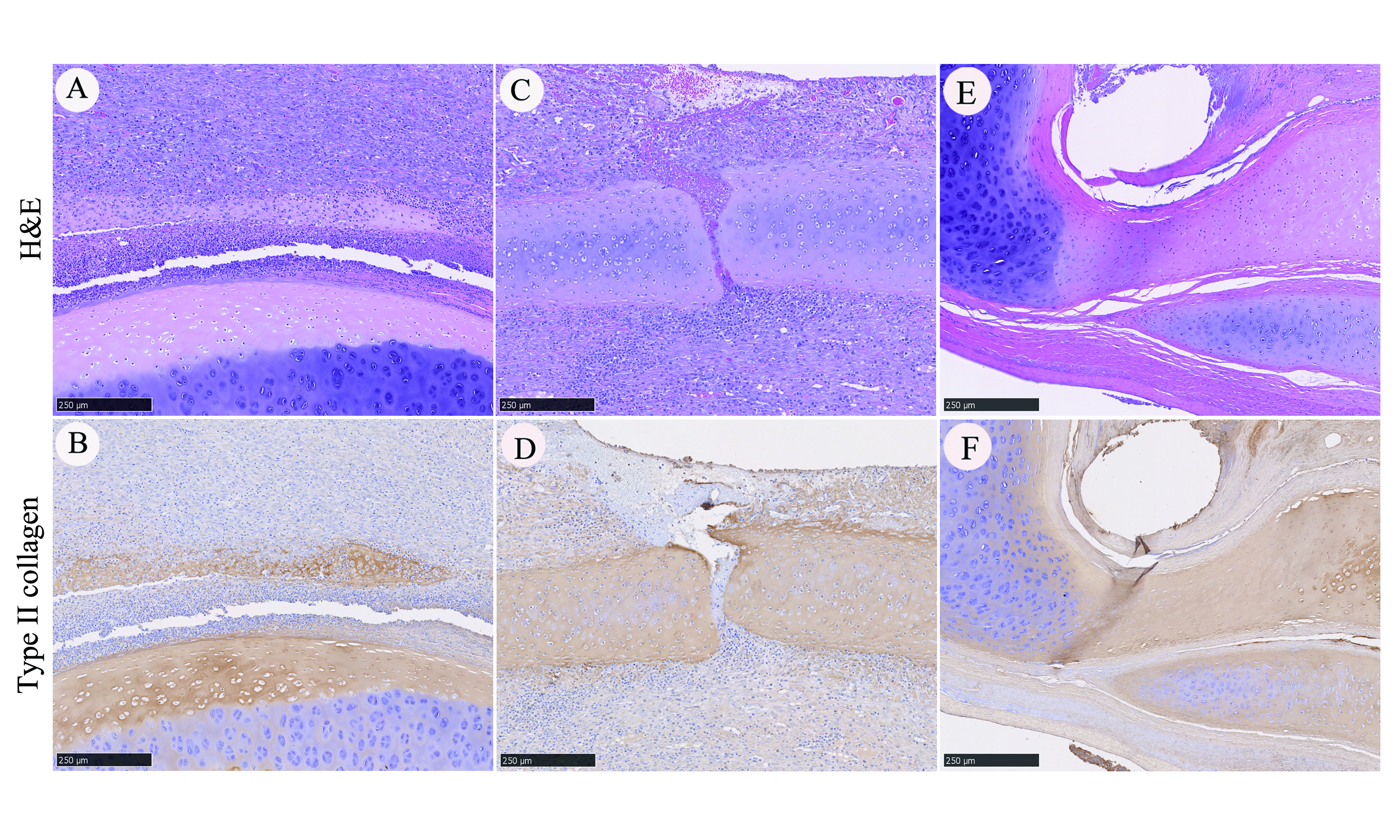


**Figure S8**. Types of Cartilage Evolution During Chondrogenesis. (A-B) Tapered spear-like neocartilage showing elongation within the trachea. (C-D) Cartilage templates ready to merge. (E-F) Fused cartilage templates. Type II collagen protein expression is indicated in brown. Images are magnified at 100X.

**Table S1.** Features of proposed 4-stage chondrogenesis in neocartilage.

| **Protein expression** | **Stage I (n=4)** | | | **Stage II (n=4)** | | | | **Stage III (n=4)** | | | | **Stage IV^#^ (n=4)** | | | | ***p value*** |
| --- | --- | --- | --- | --- | --- | --- | --- | --- | --- | --- | --- | --- | --- | --- | --- | --- |
|  | **mean±SD** | | **IQR** | **mean±SD** | | **IQR** | | **mean±SD** | | **IQR** | | **mean±SD** | | **IQR** | |  |
| **Sox9 (%)** | 97.06±0.59 | | 96.56-97.67 | 66.40±5.33 | | 61.57-71.66 | | 10.34±2.38 | | 7.99-12.56 | | 16.15±0.82 | | 15.46-17 | | *0.001*** |
| **Aggrecan (%)** | 97.19±1.66 | | 95.49-98.14 | 69.04±7.85 | | 62.22-76.92 | | 65.02±7.75 | | 57.72-72.19 | | 57.36±7.72 | | 49.66-64.17 | | *0.018** |
| **PCNA (%)** | 85.60±7.49 | | 77.8-91.78 | 45.71±9.77 | | 35.59-52.42 | | 20.22±7.10 | | 13.04-25.75 | | 13.73±3.36 | | 10.48-16.93 | | *0.002*** |
| **Alcian blue** | ++ | | | +++ | | | |  | | | | ++++ | | | |  |
| **Safranin O / fast green** | + | | | ++ | | | |  | | | | ++++ | | | |  |
| **Structures** |  |  |  |  |  | |  |  |  | |  |  |  | |  |  |
| **Chondrocytes** | 173.25±14.31 | | 158.5-182.25 | 115.50±7.55 | | 110-123.5 | | 53.75±5.32 | | 48.75-58.5 | | 51.00±1.41 | | 50-52.5 | | 0.001** |
| **Perichondrium^@^** | No | | | No | | | | Yes | | | | Yes | | | |  |
| **VCs, PPs & PRLs (interim)** | No | | | No | | | | Yes | | | | No | | | |  |
| Kruskal Wallis test. **p<0.05, **p<0.01*.  ^#^ 6-month-old porcine native trachea as baseline of Stage V.  ^@^ perichondrium contain chondroblasts and chondro-progenitor cells.  Staining index: Intense (++++); High (+++); Moderate (++); Low (+).  No: non-existence; Yes: existence. | | | | | | | | | | | | | | | | |
